# Cognitive Overload by Algorithm: Simulation Showed AI Is Not Ready to Lead Diabetic Ketoacidosis

**DOI:** 10.1002/dmrr.70144

**Published:** 2026-02-22

**Authors:** Alice Monzani, Federico Lorenzo Barra, Andrea Enzo Scaramuzza, Ivana Rabbone

**Affiliations:** ^1^ SIMNOVA‐Interdepartmental Center for Innovative Learning and Simulation in Medicine and Allied Health Professions University of Piemonte Orientale Novara Italy; ^2^ Division of Pediatrics Department of Health Sciences University of Piemonte Orientale Novara Italy; ^3^ Department of Anesthesiology and Intensive Care Medicine Azienda Ospedaliero‐Universitaria ‘Maggiore Della Carità’ Novara Italy; ^4^ Division of Pediatrics Pediatric Diabetes Endocrinology and Nutrition Azienda Socio Sanitaria Territoriale (ASST) Cremona Cremona Italy

The integration of artificial intelligence (AI) into medicine is at a critical juncture. A wave of optimism heralds its potential to revolutionise diagnostics and personalise treatment [1, 2]. Yet, as we move from using AI for discrete analytical tasks into the dynamic, high‐stakes arena of acute clinical care, a critical question remains unanswered: how does AI perform as an active team member in a crisis? In a human‐AI team, who should lead?

For diabetologists, few scenarios are more dynamic than managing paediatric diabetic ketoacidosis (DKA), a metabolic emergency requiring meticulously orchestrated interventions. This complex reality serves as an ideal testbed for the limits of clinical AI. To move beyond speculation, we must rigorously stress‐test these human‐AI team dynamics before widespread clinical deployment.

## 1 A Stress Test in a Simulated Crisis

To investigate these dynamics, we conducted a high‐fidelity simulation study. Our objective was to rigorously evaluate human‐AI team dynamics in a controlled, high‐stress environment, assessing both objective performance and subjective user experience. We tasked 12 expert paediatric diabetologists with managing a standardised paediatric diabetic ketoacidosis (DKA) case [3]. To assist in clinical decision‐making, each team used an AI chatbot powered by OpenAI's GPT‐4, enhanced with the latest DKA clinical guidelines and calculation capabilities [4, 5]. Following each session, participants completed the validated User Experience Questionnaire (UEQ) [6]. Our selection criteria for the ‘expert component’ were explicit: participants were 12 expert paediatric diabetologists, all board‐certified, with an average of 10 ± 5 years of experience in diabetes care. They were purposively recruited from tertiary academic centres known for their extensive experience in managing acute DKA in children. This ensured a high level of clinical expertise to truly stress‐test the AI's utility in real‐world scenarios. Each expert served as the team leader in a single‐blinded, standardised DKA simulation scenario designed to mimic the complexities of acute. All of them have been active members of the Italian DKA management in paediatrics guidelines working group. The AI chatbot was integrated as a continuous decision‐support tool, available throughout the simulation.

Beyond the UEQ, our study incorporated a structured qualitative synthesis of the expert component. This involved meticulous observation of team interactions during the simulation, followed by structured debriefings with each expert. During these debriefings, participants were prompted with standardised questions to elaborate on specific AI interactions, perceived cognitive load, trust in AI recommendations, and overall workflow impact. These qualitative data were systematically transcribed and analysed for recurring themes, providing rich context to the quantitative UEQ scores.

**FIGURE 1:**
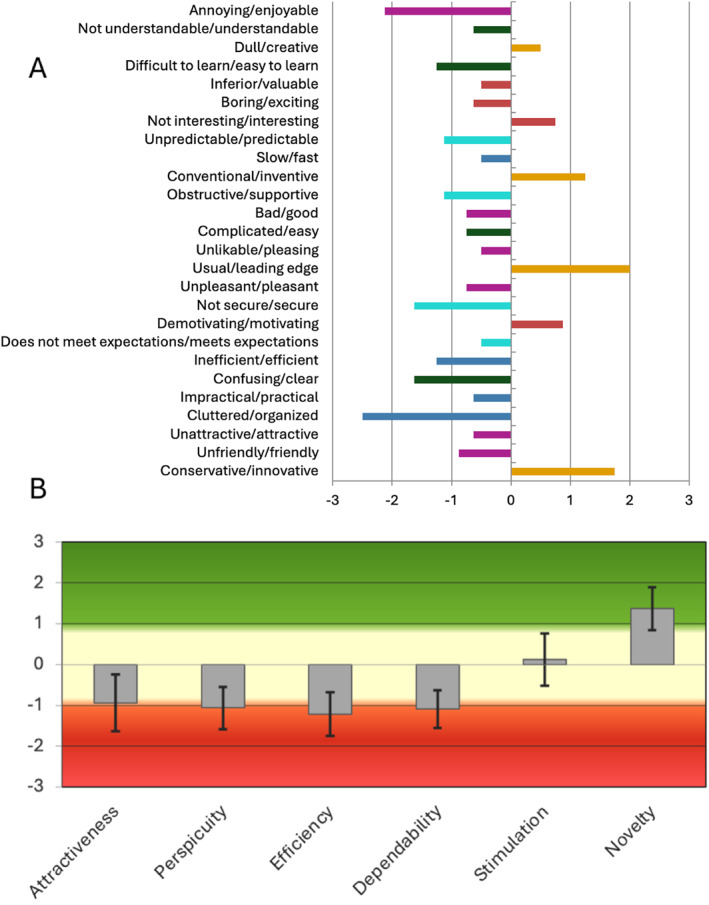
(A) Mean user experience questionnaire (UEQ) item values on a −3 to +3 scale. The figure displays the mean rating for each of the 26 UEQ items, showing a consistently negative user experience for most pragmatic and hedonic qualities of the AI tool. (B) Mean and variance across the six UEQ dimensions. The figure shows the mean scores for the six core dimensions of user experience. Scores below −0.8 indicate a negative evaluation. The AI was rated negatively on attractiveness, perspicuity, efficiency, and dependability.

Our findings reveal a stark paradox: while the AI was rated positively for ‘Novelty’ (mean + 1.4, 95% CI: +0.8 to +2.0), it scored negatively across all pragmatic dimensions critical to clinical utility: Attractiveness (mean −0.9, 95% CI: −1.3 to −0.5), Perspicuity/clarity (mean −1.05, 95% CI: −1.5 to −0.6), Efficiency (mean −1.2, 95% CI: −1.6 to −0.8), and Dependability (mean −1.1, 95% CI: −1.5 to −0.7) (Figure 1). Rather than reducing cognitive load, the AI paradoxically increased it. Expert clinicians—those most capable of inputting the precise, time‐stamped data AI requires—found the AI to be a dependent calculator, not a guide. Its verbose, context‐poor recommendations required active ‘filtering’ of suggestions and verification of outputs at the exact moment when clinical bandwidth was most constrained.

## 2 Deconstructing the Failure: The Paradox of Expertise

The AI failed not because of a knowledge deficit—it could recite the guidelines correctly—but because of a profound context deficit. An AI tool for DKA management requires a continuous feed of highly specific, time‐stamped, and error‐free data. A clinical team with the expertise to collect, verify, and input this dataset in real‐time arguably already possesses the core competencies to manage DKA. AI becomes less of a guide and more of a sophisticated but dependent calculator, its value contingent on the very human expertise it is meant to support [7, 8].

This is amplified by the ‘garbage in, garbage out’ principle [9, 10, 11]. An AI operates on the data it is given; it cannot independently question if a surprisingly low potassium value is genuine or the result of a haemolysed sample. It cannot recognise that a parent's observation about their child's baseline is invaluable data no algorithm possesses. Our qualitative synthesis strongly reinforced this; experts consistently reported the AI's inability to adapt to non‐standard presentations, integrate subtle clinical cues (e.g., patient affect, subtle changes in breathing), or prioritise information based on patient‐specific context as major impediments to its utility and drivers of increased cognitive load.

## 3 Lessons for Safer AI Deployment

Our experiment does not suggest that AI is incapable, but that real‐world tests are the only way to identify its gaps and guide its development. We propose three pragmatic rules for safer AI deployment in acute diabetes care.

1. Mandate rigorous simulation testing: High‐fidelity simulation is not a luxury but an essential diagnostic tool. It allows us to identify AI's failure points—automation bias, workflow disruption, cognitive overload—in a safe environment before they can impact patient care. Regulatory bodies and healthcare systems should consider simulation‐based validation a prerequisite for deploying AI in high‐stakes clinical roles.
2. Clarify roles: the AI as a specialised co‐pilot: The goal should not be to create an AI leader. Instead, we must design AI for specific, subordinate roles where it excels: calculating fluid rates, flagging lab abnormalities, or serving as an interactive checklist. Complex, context‐sensitive decisions and strategic oversight must remain firmly in human hands. The AI should be a co‐pilot that provides data and performs calculations, not the captain charting the course.
3. Build for trust and literacy: For any human‐AI team to function, clinicians must trust the tool. This requires building ‘explainable AI’ (XAI) that clarifies its reasoning, not just its recommendations [9]. Concurrently, medical education must evolve to include AI literacy, equipping clinicians to use these tools effectively, recognise their limitations, and override them with confidence when their clinical judgement dictates [12].

## 4 Conclusion

Our simulation serves as a blueprint for AI's safe integration into clinical care: test, fail, analyse, and refine. It demonstrates that for high‐stakes scenarios like DKA, the immediate value of AI lies not in clinical leadership, but in acting as a well‐defined decision‐support aid [13].

In the complex choreography of managing a critically ill child, the human clinician must remain the orchestrator, the critical thinker, and the ultimate decision‐maker. Until AI can irrefutably demonstrate safety, contextual understanding, and a proven ability to reduce—not increase—the cognitive burden on clinicians, the captain of the clinical ship must unequivocally be human.

## Funding

The authors have nothing to report.

## Conflicts of Interest

The authors declare no conflicts of interest.

## Data Availability

The data that support the findings of this study are available on request from the corresponding author. The data are not publicly available due to privacy or ethical restrictions.
